# Microplastic Contamination of the Turkish Worm Lizard (*Blanus strauchi* Bedriaga, 1884) in Muğla Province (Türkiye)

**DOI:** 10.3390/biology14040441

**Published:** 2025-04-19

**Authors:** Cantekin Dursun, Nagihan Demirci, Kamil Candan, Elif Yıldırım Caynak, Yusuf Kumlutaş, Çetin Ilgaz, Serkan Gül

**Affiliations:** 1 Department of Biology, Faculty of Arts and Sciences, Recep Tayyip Erdoğan University, 53100 Rize, Türkiye; cantekin.dursun@erdogan.edu.tr (C.D.); nagihan_demirci19@erdogan.edu.tr (N.D.); 2 Department of Biology, Faculty of Science, Dokuz Eylül University, Buca, 35390 İzmir, Türkiye; kamil.candan@deu.edu.tr (K.C.); yildirim.elif@deu.edu.tr (E.Y.C.); yusuf.kumlutas@deu.edu.tr (Y.K.); cetin.ilgaz@deu.edu.tr (Ç.I.); 3 Fauna and Flora Research and Application Center, Dokuz Eylül University, Buca, 35390 İzmir, Türkiye

**Keywords:** food web, GITs, microplastic, reptiles, pollution, ecological risk

## Abstract

Because of their diversity, microplastics (MPs), which are synthetic particles smaller than 5 mm, are highly bioavailable and widely distributed. The prevalence of microplastics in aquatic habitats has been extensively studied but less is known about their presence in terrestrial environments and biota. This study examined MP intake in terrestrial environments utilizing gastrointestinal tracts (GITs), with a particular focus on the Turkish worm lizard (*Blanus strauchi*). Suspected particles discovered in the GITs were removed, measured, and characterized based on size, shape, color, and polymer type in order to evaluate MP ingestion. Out of 118 samples analyzed, 29 specimens (or 24.57%) had microplastic particlesMP length did not significantly correlate with snout–vent length (SVL) and weight. These correlations were tested to determine whether the size or weight of *Blanus strauchi* influenced the amount or size of MPs found within the GITs. Also, MP consumption by the worm lizard did not correlate with the year of sampling. All particles identified as fibers through FT-IR spectroscopy analysis. The most common type of microplastic was polyethylene terephthalate (PET). The most often detected color was blue, with mean MP lengths ranging from 133 µm to 2929 µm. It has been demonstrated that worm lizards inhabiting soil or sheltering under stones in bushy areas with sparse vegetation consume MPs. Predation is regarded to be the most likely way through which MPs infiltrate terrestrial food webs.

## Simple Summary

Although microplastics (MPs) are small, artificial particles that can be found in a variety of settings, little is known about how they affect species that live on land. Through gastrointestinal tract analysis, this study examined MP consumption in the Turkish worm lizard (*Blanus strauchi*). Of the 118 specimens analyzed, 24.57% had microplastics; the only kind identified was fibers, which are mostly made of polyethylene terephthalate (PET). The majority of the particles were blue, and their diameters varied from 133 µm to 2929 µm. According to this study, MPs can enter terrestrial food webs because these lizards, who live in soil and under stones, ingest them most likely through predation.

## 1. Introduction

MPs are plastic pieces smaller than 5 mm that are formed through the breakdown of plastic materials such as plastic bags, bottles and packaging that are thrown into the environment over time. MPs, which are divided into two groups as primary and secondary, are introduced into terrestrial and aquatic ecosystems through various means such as textile products, cosmetics, cleaning materials and the breakdown of large plastics [1,2,3]. These MPs, which consist of different types of plastic such as polyethylene, polypropylene, polyester and polyurethane, not only cause environmental pollution but also threaten ecosystems, reduce biodiversity, and pose potential threats to the health of living organisms [1,4,5]. Many living species can easily ingest microplastics and take them into their bodies. In addition, MPs that enter the digestive system can pass into the circulatory system and tissues, causing infections and necrosis in affected areas [6,7,8]. These strengthen concerns that MPs can harm both human health and ecosystems [9,10].

Reptiles are ectodermic animals and are highly sensitive to changing environmental conditions. They depend on their environment to regulate their body temperature and metabolic processes, as well as for reproduction and development. Reptiles, which can live in freshwater, marine and terrestrial habitats, are exposed to pollutants in various biota [11]. It has been recorded in studies that aquatic reptiles can become entangled in plastic fishing nets [12] and may swallow plastic caps [13], and that the presence of plastic has also been detected in terrestrial reptiles [14,15], and that proximity to water sources has no effect on microplastic ingestion [16]. Studies show that MPs can negatively affect the physiological functions, reproduction, and survival abilities of reptiles [17,18,19]. Lizards, which belong to the reptile class, are considered good bio-monitors for assessing environmental pollution. One of the main reasons for this is that lizards can live in small areas and associate with a specific habitat. In addition, lizards are sensitive to environmental change. They may show physiological and behavioral changes against factors such as pesticide exposure and environmental degradation [20,21,22,23].

Microplastic studies have been carried out on many animal species [24,25,26,27]. Compared to these species, studies microplastic ingestion in reptiles are more limited [15,19]. Therefore, the aims of this study are (1) to assess the ingestion of microplastics by a Turkish worm lizard (*Blanus strauchi*), one of the amphisbaenian species, (2) to characterize and quantify the particles found in gastrointestinal tracts (GITs) of *Blanus strauchi*, and (3) to monitor anthropogenic pollution sources in a touristic region. Due to the burrowing habits of *B. strauchi*, it is in close contact with the soil substrate, which is a potential site for microplastic accumulation and persistence. Unlike many surface-dwelling species, *B. strauchi* is continually exposed to MPs buried in the ground, so it is a sensitive indicator of terrestrial plastic pollution. As a predator of soil invertebrates, trophic transfer may also indirectly devour MPs. Due to restricted habitat and sensitivity of the species to environmental changes, *B. strauchi* fairly depicts local pollution levels. Ecological and physiological traits of *B. strauchi* make it a great indicator species for estimating MPs in soil-based food webs, particularly in regions impacted by human activities like tourism [28,29,30].

## 2. Materials and Methods

### 2.1. Studied Species

**Figure 1:**
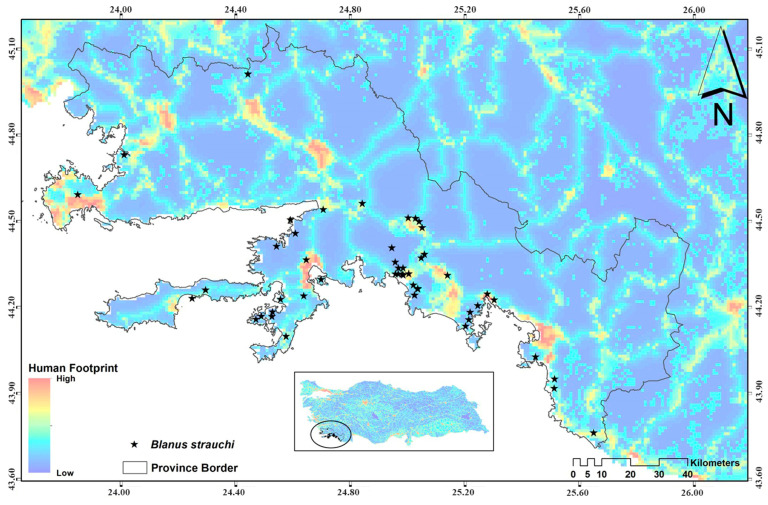
Human footprint map showing the impact of direct pressures from human activities on nature between 1993 and 2009 (from [31]). Dots show the distribution localities of *Blanus strauchi*.

The Turkish worm lizard is a limbless reptile species belonging to the family Blanidae. In the European region, this species occurs in Rhodes, Kos, Kalymnos and other southeastern Aegean islands of Greece. From there, it spreads to western and southern Anatolian Türkiye [28] (Figure 1). This species lives predominantly in scrubland habitats and develops at altitudes ranging from sea level to approximately 1400 m. Its burrowing nature allows it to navigate and inhabit loose, sandy, or soft soil substrates [29]. Being a carnivorous species, *B. strauchi* primarily preys on small invertebrates. Its diet largely consists of insects such as ants, spiders, and beetles [30]. A total of 118 *Blanus strauchi* individuals were obtained from the Fauna and Flora Research and Application Center at Dokuz Eylül University (Table S1). These specimens were collected from small islands in and around Muğla province (Figure 1) and preserved in the museum’s collections in glass jars that were filled with 96% ethanol. Also, the sampling was conducted eight times over 32 years. The human footprint map was classified into four categories using ArcMap v10.4.1, and the samples within this class were categorized as low, medium, high and very high anthropogenic impact (Table S1).For the first measurements, a digital caliper Mitutoya 500-161-30 (Ontario, Canada) was used to precisely measure each specimen’s body length to the nearest 0.01 mm from the snout tip to the cloak on steel tray covered with wax to stretch the specimens. Following that, the GITs of each sample were meticulously removed from the ventral section using a scalpel. The upper esophagus and anal orifice were then cut to acquire the gastrointestinal tract as a whole in order to study MPs. A balance Ohaus EX223N (Ontario, Canada) was used to determine each sample’s GIT weight with a precision of 0.01 g. Following this, all GITs were meticulously stored in glass jars filled with 96% ethanol (Merck; Darmstadt, Germany) for later use in this study.

### 2.2. Studied Province

Muğla province, located in southwest Turkey, is a popular tourist destination due to its extensive coastline, rich cultural legacy, and stunning natural beauty. The region includes well-known tourist sites such as Bodrum, Marmaris, Fethiye, and Datça, which draw millions of domestic and international visitors each year. Muğla’s tourism industry mostly focuses on seaside and resort tourism, but ecotourism, cultural tourism, and rural tourism are also gaining popularity [32]. The region’s tourism industry is largely seasonal, which frequently results in environmental challenges such as shoreline erosion, water resource misuse, and habitat loss, particularly during the summer months (available at http://www.mugla.gov.tr (20 March 2025)).

### 2.3. Characterization of Microplastics

MPs were extracted from the gastrointestinal tracts through the consumption of tissue-corrosive reactants, such as hydrogen peroxide (H_2_O_2_, 30% *w*/*w*). Strong oxidizing agents like H_2_O_2_ are utilized extensively as digesting agents to extract microplastic particles in animals’ gastrointestinal tracts [33,34]. Glass cylinder tubes of 25 cm in length and 2.5 cm in diameter were filled with pre-weighed tissues for the digesting process. The tubes were then placed in a batch reactor that was set to 65 °C. During the investigation, each tube was covered with a watch glass and filled with 5 mL of H_2_O_2_ aqueous solution per gram of tissue. Ultimately, the contents of the tube were collected using vacuum filtration on Whatman Grade 4 qualitative filter paper with particle sizes of 20–25 µm. The papers were then placed in glass Petri dishes for examination under a microscope.

**Figure 2:**
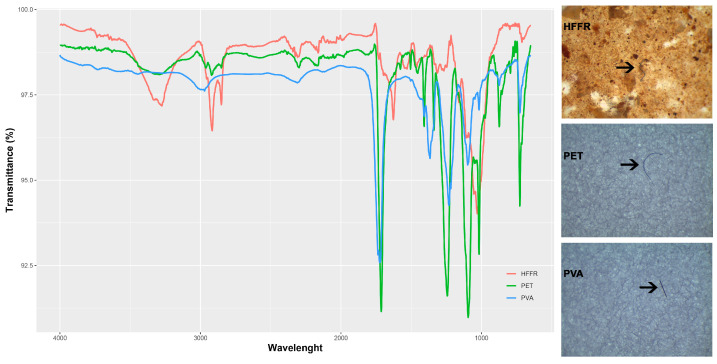
Representative FT-IR spectrum of identified microplastic types (PET: polyethylene terephthalate; HFFR: Halogen Free Flame Retardant; PVA: Polyvinyl alcohol). Arrows on plastic figures show items presented in spectra.

Using a Leica S6D^®^ stereomicroscope (Wetzlar, Germany), filtered samples suspected to be MPs were examined, and they were categorized based on physical characteristics like color and shape. In order to characterize the potential MPs under regular operating conditions, a Fourier Transform Infrared spectrometer FT-IR, PerkinElmer Spectrum 100 (PerkinElmer, Waltham, MA, USA) with an attenuated total reflectance (ATR) apparatus was used. The spectral range was 4000–650 cm^−1^, with 12 repeated scans at a resolution of 2 cm^−1^. Each candidate was given a similarity score by comparing the examined spectra with the distinctive FT-IR signals of the reference polymer in the PerkinElmer SEARCH Plus ATR Polymer library. As a result of comparing the analysis results with the library data, a standard spectrum comparison was made for each sample, taking the percentage values provided by the program as reference. Thus, a similarity threshold of 70% was then used to identify the type of polymer (Figure 2). Using ImageJ software v1.46r and ruler calibration based on 1 mm under × 4 magnification, a size analysis of MPs was performed on MP pictures captured using a stereomicroscope [35]. Thus, each microplastic’s length was measured. To protect against contamination, pure cotton laboratory coats and disposable nitrile gloves were used. All tools and equipment were made of glass and steel, and thoroughly washed with distilled water before use.

### 2.4. Statistical Analyses

Descriptive statistics were computed with the *psych* package v2.2.5 [36], and the normality assumption was checked using the *olsrr* package v0.5.3 [37] for all continuous variables. The relationships between microplastic length, SVL, body weight, and GIT weight were investigated using correlation and regression analyses with the *stats* package v4.1.2 [38]. To demonstrate the frequency of identified microplastic characteristics and compare the proportional contributions across microplastic characteristics, pie-donut charts were constructed using the *webr* package v0.1.6 [39]. Additionally, an alluvial diagram was created to demonstrate the flow among microplastic characteristics using the package *ggalluvial* v0.12.5 [40]. Multiple comparisons of length difference among color categories were shown with boxplots using the package *ggpubr* v0.4.0 [41]. Lastly, the Chi-square test was used to interpret the difference between microplastic color and year. All analyses were executed in R Programming Language v4.1.2 [38].

## 3. Results

**Figure 3:**
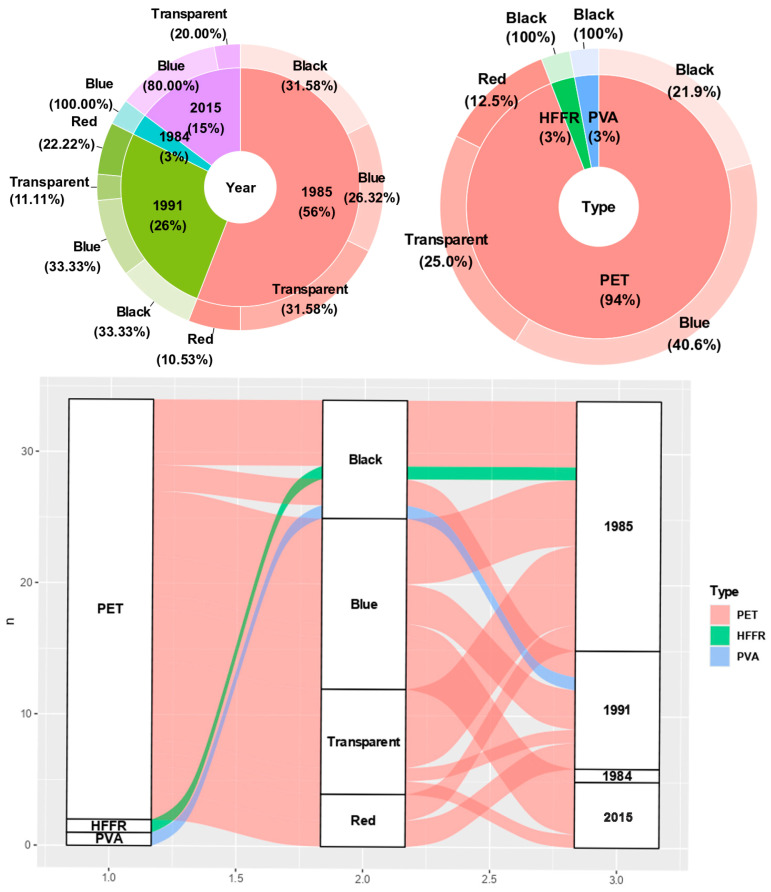
Pie-donut chart plots demonstrating color percentages in year and type, as well as alluvial plot showing the flow between MP categories.

MPs were detected in the GITs of 29 specimens, representing 24.57% of all samples (N = 118). A total of 34 distinct particles were identified, corresponding to an average of 1.17 particles per individual with MPs, and 0.28 particles per individual across all examined samples. The highest abundance of MPs was recorded from Sedir Island, Marmaris (N = 4), accounting for 11.76% of the total identified MPs. In terms of shape, only fibers were detected (100%). Regarding particle type, polyethylene terephthalate (PET) was the predominant material, accounting for 94.12% of the particles (N = 32), followed by one high-density flexible foam rubber (HFFR) particle (2.94%) and one polyvinyl alcohol (PVA) particle (2.94%; Figure 3).

**Figure 4:**
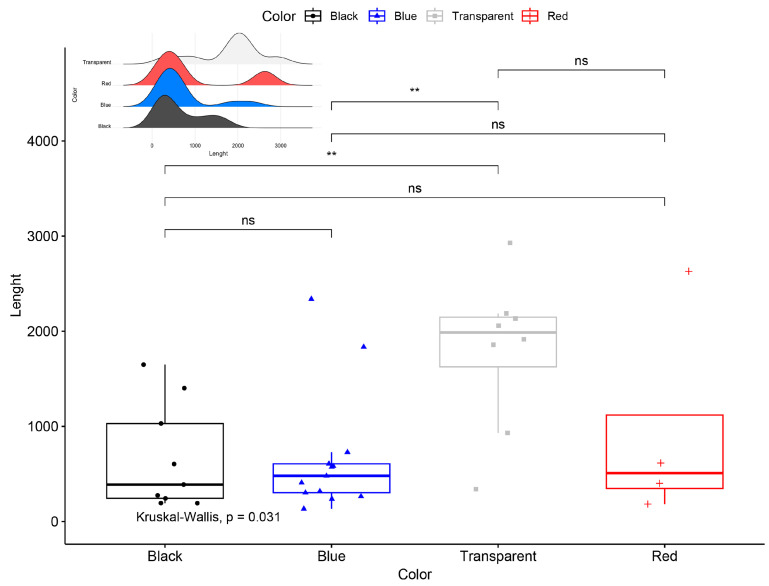
Box plot of the Kruskal–Wallis test for values of the MP length by color. The inset figure indicates the density of the size distribution. **: *p* < 0.01, ns: non-significant difference.

The most common color observed was blue (N = 13), followed by black (N = 9), transparent (N = 8), and red (N = 4). Transparent fibers exhibited a knotty, mass-like shape in some instances and were significantly larger than black and blue fibers in terms of mean MP length (Figure 4). The mean MP lengths were as follows: 1794.00 ± 283.65 µm for transparent fibers, 957.25 ± 564.51 µm for red fibers, 667.31 ± 182.01 µm for blue fibers, and 664.00 ± 186.41 µm for black fibers. The overall mean length of all MPs (N = 34) was 969.47 ± 144.37 µm, with sizes ranging from 133 µm to 2929 µm. The observed MPs spanned 32 years, with the majority of plastics originating from 1985 (N = 19), followed by 1991 (N = 9), 2015 (N = 5), and 1984 (N = 1). Plastics from 1985 represented 56% of the total observations (N = 19). Additionally, six black and transparent fibers were found from this period. However, a Chi-square test revealed no significant difference in the color distribution of MPs across different years (χ^2^ = 8.91; df = 9; *p* > 0.05).

The correlation test revealed a significant relationship between SVL and weight (r = 0.77, *p* < 0.001), SVL and GIT weight (r = 0.37, *p* < 0.01), and weight and GIT weight (r = 0.64, *p* < 0.001). The mean SVL was 17.27 ± 0.24 cm (range: 14.80–19.80 cm), the mean weight was 6.56 ± 0.25 g (range: 3.50–8.90 g), and the mean GIT weight was 0.52 ± 0.06 g (range: 0.10–1.46 g). Linear regression models also verified the correlation results for SVL–weight (F = 48.78; R2: 0.60; *p* < 0.001), SVL–GIT weight (F = 5.30; R2: 0.11; *p* < 0.05), and weight–GIT weight (F = 22.74; R2: 0.41; *p* < 0.001). However, MP length did not show a significant correlation with any variable (*p* > 0.05). Moreover, there was not a significant correlation between the presence of MPs and SVL (r = −0.11; *p* > 0.05) as well as the presence of MPs and weight (r = 0.21; *p* > 0.05).

## 4. Discussion

Although MPs have primarily been monitored in aquatic environments [42,43,44,45], increasing evidence indicates their widespread presence in terrestrial ecosystems, including soil environments [46,47,48,49]. MPs can enter the soil through various pathways, such as atmospheric deposition, human activities, water usage, and agricultural practices [50,51,52]. Notably, MP pollution in agricultural lands has been reported to be up to 23 times higher than in aquatic environments [53]. In this study, the presence of MPs was monitored for the first time in *Blanus strauchi* (Turkish worm lizard), a reptile adapted to a subterranean lifestyle. To date, MP contamination has primarily been documented in marine reptiles, such as sea turtles [54,55,56,57], as well as in terrestrial lizards, geckos [15,58,59,60,61], and snakes [14]. However, no prior reports exist for any Amphisbaenian species.

MPs were detected in nearly a quarter of all samples investigated in this study. In reptiles, Dursun et al. [15] reported MPs in 25 out of 300 *Ophisops elegans* samples, corresponding to 8.33% of individuals. In a study on six reptile species from Thailand, Teampanpong and Duengkae [60] observed an MP presence rate of 71.43% (five out of seven samples). Similarly, MP occurrence ranged from 33.30% to 66.60% in feces and 66.60% to 75.00% in the gastrointestinal tracts (GITs) of two *Hemidactylus* species from Cuba [61]. Mackenzie and Vladimirova [21] recorded MP occurrence rates of 12.03% in 133 samples of *Hemidactylus mabouia* and 6.00% in 50 samples of *Tropidurus torquatus* from South Paraguay. MP prevalence varies across reptile species. This variation is likely to be influenced more by environmental factors and the extent of MP pollution than by species-specific characteristics. However, significantly higher ingestion rates have been reported in marine reptiles compared to terrestrial species. For instance, *Caretta caretta* individuals were found to ingest an average of 7.94 items [62] and 49.24 items [63], while Jung et al. [64] documented an average of 63.20 items per individual across three *Caretta* species. This suggests that body size may play a crucial role in the accumulation of MPs within the gastrointestinal tract.

In this study, all detected MPs were in fiber form. Due to their widespread presence in both terrestrial and aquatic environments, fiber MPs have been increasingly reported in various animal groups, ranging from invertebrates to vertebrates [27,65,66,67,68,69,70,71]. Fibers primarily originate from the degradation of larger plastic materials and synthetic textile products, entering ecosystems through agricultural activities, wastewater discharge, and atmospheric deposition [25,72]. Furthermore, they can be transferred from marine to terrestrial organisms via trophic bioaccumulation [73]. Given that fibers are the most commonly reported plastic type in environmental monitoring studies, the findings of this study align with existing literature.

Three different MP types were detected in this study, with polyethylene terephthalate (PET) being the most prevalent polymer, followed by one halogen-free flame retardant (HFFR) and one polyvinyl alcohol (PVA) item. PET is a widely used polymer in industrial production, commonly associated with textile manufacturing, water bottles, and food packaging. HFFRs are primarily used in safety applications for electrical devices, cables, and construction materials, while PVA is a water-soluble polymer frequently utilized in packaging, medical applications, textiles, and the paper industry [40,74]. Previous studies have similarly identified PET as the dominant polymer in various organisms. For instance, Dursun et al. [15] reported that PET constituted 54.06% of all monitored MPs in the gastrointestinal tracts (GITs) of *Ophisops elegans*. The authors also suggested that PET’s resistance to degradation allows it to persist in the environment as small microfiber particles, increasing the likelihood of ingestion or transfer between organisms. Prata et al. [75] investigated MPs in the internal tissues of animals from urban areas and found a high prevalence of small-sized PET particles. Similarly, Dursun et al. [76] examined MP contamination in *Neurergus* species from Türkiye, reporting PET as the most abundant polymer (63.63%, N = 44). While PET is widely documented in MP studies, other polymers, such as PVA, have been less frequently reported in animal samples. PVA presence has primarily been noted in fish species [77,78].

In terms of MP color, blue and transparent fibers accounted for 72.41% of all detected items, with these colors corresponding to the longest fibers. Such fibers predominantly originate from synthetic materials used in clothing, textiles, fishing nets, water bottles, and woven sacks. Similar color distributions have been reported in previous studies. For example, Dursun et al. [79] analyzed MP contamination in *Pelophylax* species and found that 81.34% of MPs were either blue or transparent. Deoniziak et al. [80] investigated MP pollution in terrestrial environments using dead birds from the genus Turdus and identified transparent particles as the dominant type, comprising 75.00% of all MPs. These findings suggest that blue and transparent MPs serve as direct indicators of anthropogenic pollution in animals. However, pollution sources infiltrating the environment via main sources, including microbeads in cosmetics, synthetic textile fibers, and industrial abrasives, alongside secondary sources such as the degradation of bigger plastic trash, tire wear, deterioration of fishing gear, and effluent from wastewater treatment facilities influence color variation. Therefore, the absence of a statistically significant difference in MP colors may indicate that multiple environmental factors contribute to MP distribution.

MPs are now recognized as widespread pollutants across terrestrial and aquatic environments. However, subterranean ecosystems—such as groundwater systems, cave networks, and subsoil habitats—have not been studied as extensively. Although emerging evidence confirms the presence of microplastics in these environments, such findings are still relatively limited [81,82,83]. This knowledge gap can be attributed to the difficulty of accessing subterranean sites and the long-standing assumption that these systems are less exposed to surface contaminants. From another perspective, animals living in caves, karstic areas, or under soil are poorly investigated in terms of MP ingestion and there is limited literature on them [84,85]. MPs can penetrate soil environments through a variety of pathways. Sources such as sewage sludge and compost derived from municipal waste, which often contain high concentrations of microplastics, can introduce these particles into the soil. Besides agricultural practices, such as the use of plastic mulching films, irrigation with contaminated water, and the application of organic fertilizers, can lead to soil contamination. Once in the soil, these particles can be transported vertically and horizontally through bioturbation, percolating water, and soil fauna, such as earthworms and insects, potentially reaching deeper layers and even groundwater systems. Therefore, finding MPs in *B. strauchi* probably derived from due either to agricultural activities or to plastic debris associated with extensive tourism in Muğla. Given that numerous insect and larval shells were found in GIT contents, ingestion of MPs can be relevant to feeding habits, as an alternative scenario.

## 5. Conclusions

This study represents the first report of microplastic contamination in *Blanus strauchi*, highlighting the presence of MPs in soil environments. The similarity in MP occurrence between *Blanus strauchi* and other terrestrial and marine reptiles further underscores the pervasiveness of MP contamination across ecosystems. Future studies should further explore the long-term ecological impacts of MP accumulation in reptiles, particularly those adapted to subterranean habitats, and assess the potential for bioaccumulation and trophic transfer within and across ecosystems.

## Data Availability

The original contributions presented in this study are included in the article. Further inquiries can be directed to the corresponding authors.

## Supplementary Materials

The following supporting information can be downloaded at: https://www.mdpi.com/article/10.3390/biology14040441/s1, Table S1: The information of the museum material samples used in the study.
