# Supplementary material for: Microplastic Contamination of the Turkish Worm Lizard (Blanus strauchi Bedriaga, 1884) in Muğla Province (Türkiye)
Source: Biology (Basel). 2025 Apr 19;14(4):441. doi: 10.3390/biology14040441 (PMC12025114; doi:10.3390/biology14040441)
Supplement: Supplementary file 1 [file biology-14-00441-s001.zip › biology-3584023-supplementary.pdf]

Article

Microplastic Contamination of the Turkish Worm Lizard (*Blanus strauchi* Bedriaga, 1884) in Muğla Province (Türkiye)

Cantekin Dursun [1], Nagihan Demirci [1], Kamil Candan [2,3], Elif Yıldırım Caynak [2,3], Yusuf Kumlutaş [2,3], Çetin Ilgaz [2,3] and Serkan Gül [1,*]

1 Department of Biology, Faculty of Arts and Sciences, Recep Tayyip Erdoğan University, 53100 Rize, Türkiye; cantekin.dursun@erdogan.edu.tr (C.D.); nagihan_demirci19@erdogan.edu.tr (N.D.)
2 Department of Biology, Faculty of Science, Dokuz Eylül University, Buca, 35390 İzmir, Türkiye; kamil.candan@deu.edu.tr (K.C.); yildirim.elif@deu.edu.tr (E.Y.C.); yusuf.kumlutas@deu.edu.tr (Y.K.); cetin.ilgaz@deu.edu.tr (Ç.I.)
3 Fauna and Flora Research and Application Center, Dokuz Eylül University, Buca, 35390 İzmir, Türkiye
* Correspondence: serkan.gul@erdogan.edu.tr

Table S1. The information of the museum material samples used in the study.

CODE	SVL (cm)	BODY WEIGHT (g)	MP OCCURRENCE	LOCALITIES	ANTROPOGENIC EFFECT	MUSEUM NO	DATE	LATITUDE	LONGITUDE
B1	19.5	6.6	Present						
B2	17	7.2	Present						
B3	19.4	9.1	Absent						
B4	15.6	4.9	Present	Tersane Adası, Fethiye	Low	16/1985	13.02.1985	36.674349	28.919923
B5	16.3	6.6	Present						
B6	17.6	6.2	Absent						
B7	16.9	6.8	Absent						
B8	21.3	18.1	Absent						
B9	21.3	17.4	Absent	Kavakarası köyü, Köyceğiz	Medium	222/1991	02.05.1991	36.887430	28.720897
B10	19.7	9	Absent						
B11	20	9.2	Absent						
B12	18.5	8.2	Present	Sülüngür gölü, Dalyan, Ortaca	Low	301/1991	02.03.1991	36.793448	28.654045
B13	17.4	7.2	Present						
B14	18.2	7.3	Absent	Çadırgediği, Köyceğiz	High	328/1991	13.12.1991	36.863862	28.598886
B15	18.6	7	Absent						
B16	18.1	7.8	Absent						
B17	18.2	8.5	Present	Köyceğiz	Medium	288/1991	16.05.1991	36.999557	28.682954
B18	17.2	7.4	Absent						
B19	17.9	8.6	Absent						
B20	18.3	7.6	Present	Gökbel köyü, Ortaca	Medium	266/1991	02.03.1991	36.781281	28.668847
B21	17.2	5.5	Absent	Tepearası köyü, Köyceğiz	Low	190/1991	13.04.1991	36.876311	28.704868
B22	18.7	7.6	Absent	Köyceğiz Gölü	Medium	168/1991	01.03.1991	36.907264	28.595023
B23	19.4	8.1	Absent	Yıldız adası, Marmaris	Medium	58/1984	08.04.1984	36.810734	28.289122
B24	14.9	3.4	Absent						
B25	17.4	5.9	Absent						
B26	17.3	6.5	Present	Köyceğiz	Medium	343/1991	17.04.1991	36.998492	28.711445
B27	19.3	8.4	Present						

B28	15	4	Absent						
B29	16.8	4.5	Absent	Gökbel köyü, Ortaca	Medium	137/2013	22.04.2013	36.783381	28.669663
B30	15.5	4.2	Absent						
B31	21.6	9.9	Absent						
B32	18.3	6.6	Absent	Emecik köyü, Datça	Low	138/2013	21.04.2013	36.778679	27.820029
B33	18.2	7.8	Absent						
B34	16.9	6.3	Absent						
B35	17	6.4	Absent						
B36	16	5.8	Absent						
B37	16.5	5.6	Absent	Karacaada, Marmaris	Low	41/1985	08.04.1985	36.958602	28.191741
B38	18.8	6.9	Absent						
B39	17.4	5.4	Absent						
B40	16.8	5.2	Present						
B41	17.4	7.5	Present						
B42	17.9	7.3	Present	Sedir adası, Marmaris	High	61/1985	09.04.1985	36.991916	28.207140
B43	17.6	7.1	Absent						
B44	17.9	7.8	Present						
B45	19.6	11.5	Absent	Göcek adası, Fethiye	Medium	04/1985	12.02.1985	36.728453	28.943767
B46	16.4	5	Present						
B47	20.3	10.6	Absent	Karaağaç, Fethiye	Medium	83/2015	26.03.2015	36.476254	29.112110
B48	18.9	8.4	Absent						
B49	19.8	8.9	Present	Çandır köyü, Köyceğiz	Low	294/1991	28.03.1991	36.827595	28.602219
B50	20	9.7	Absent						
B51	17	7.3	Present						
B52	16.7	6.2	Absent	Kameriye adası, Marmaris	Medium	76/1985	11.04.1985	36.729025	28.054859
B53	17.2	6.9	Absent						
B54	18.8	8.1	Absent						
B55	15.8	3.6	Absent	Yılan adası, Kaş	Low	24/1985	14.02.1985	36.215543	29.356078
B56	17.2	4.4	Absent						
B57	15.9	5.8	Present	Kargıadası, Marmaris	Low	71/1985	10.04.1985	36.746430	28.081383

B58	17.2	6.7	Absent						
B59	18.8	7.2	Absent						
B60	17.4	7.4	Absent						
B61	18.5	8.1	Absent	Köyceğiz	Low	340/1991	19.04.1991	36.988504	28.722468
B62	20.4	9.7	Absent						
B63	17.3	5.1	Present						
B64	16.3	6.1	Present	Domuz adası, Fethiye	Low	22/1985	14.02.1985	36.662327	28.900010
B65	17.9	5.8	Absent						
B66	17.3	6.4	Absent						
B67	14.9	4.2	Absent	Hacıhalil adası, Fethiye	Low	08/1985	12.02.1985	36.696593	28.925701
B68	16.2	4.7	Absent						
B69	17	5.2	Absent						
B70	16.6	6.6	Absent	Kahya adası, Marmaris	Medium	116/1985	09.04.1985	36.944641	28.154932
B71	18	7.4	Present						
B72	18.4	7.3	Present						
B73	18.8	8.3	Absent	Gökova	High	38/1984	06.04.1984	37.025940	28.347113
B74	16.5	4.7	Absent						
B75	17.7	6	Absent						
B76	16.8	4.9	Absent	Zeytinalanı köyü, Köyceğiz	Medium	285/1991	15.03.1991	36.969105	28.730169
B77	17.3	6	Absent						
B78	18.5	7.6	Absent	Yassıada, Datça	Very high	99/1985	14.04.1985	36.752470	27.761179
B79	18	7.2	Absent						
B80	19	8.1	Absent						
B81	18.6	6.9	Absent	Marmaris	Medium	298/1996	09.03.1996	36.870620	28.244147
B82	17.7	6.8	Absent						
B83	16.2	5.8	Absent						
B84	17	6.1	Absent	Kavakarası köyü, Köyceğiz	Very high	291/1991	19.05.1991	36.887430	28.720897
B85	16.8	6.2	Absent						
B86	19	7.6	Absent	Kaunos harabeleri, Ortaca	High	160/1991	04.05.1991	36.824961	28.621775
B87	18.6	7.8	Absent						

B88	18.4	6.9	Absent						
B89	17.8	6.8	Absent						
B90	19.6	8.4	Absent	Dalyan, Ortaca	Low	296/1991	15.11.1991	36.828550	28.644209
B91	17.2	6.5	Absent						
B92	18.3	7.2	Absent						
B93	17.8	6.2	Absent	Kışla mahallesi, Dalyan, Ortaca	High	334/1991	16.03.1991	36.762287	28.652542
B94	17	5.9	Absent						
B95	16.8	5.8	Absent	Karaböğürtlen köyü, Ula	Medium	140/1986	23.04.1986	37.043275	28.508700
B96	16.8	7.1	Absent						
B97	16.1	5.7	Absent	Çandır köyü, Ortaca	High	153/1991	16.11.1991	36.826919	28.617008
B98	17	6	Absent						
B99	17.8	5.8	Absent	Delikada, Ortaca	Medium	238/1991	03.05.1991	36.796899	28.597068
B100	18.3	6	Absent						
B101	17.2	6.4	Absent	Horozlar mevki, Köyceğiz	Low	155/1991	13.12.1991	36.845584	28.625236
B102	16.7	5.7	Absent	Sarsala koyu, Dalaman	Low	25/2012	10.03.2012	36.663608	28.847385
B103	16.6	5.3	Absent	Zeytinalanı köyü, Köyceğiz	High	305/1991	17.11.1991	36.969105	28.730169
B104	15.4	4.5	Present						
B105	18.1	6.4	Absent	Kayaköy, Fethiye	Medium	147/2013	22.04.2013	36.573107	29.090535
B106	17.9	7.3	Absent	Kabirgediği Çandır köyü arası, Köyceğiz	Low	274/1991	04.05.1991	36.845588	28.607244
B107	15.8	4.4	Absent	Osmaniye köyü, Marmaris	Low	143/2013	21.04.2013	36.760095	28.209136
B108	17.6	5.7	Present	Kışla mahallesi, Dalyan, Ortaca	Medium	203/1991	14.04.1991	36.762287	28.652542
B109	15.5	4.8	Absent	Göcek, Fethiye	High	26/1985	15.02.1985	36.766344	28.943439
B110	18.4	7.4	Present	Bodrum	High	81/2015	12.04.2015	37.070669	27.375752
B111	15.9	5.4	Present	Kelebekler vadisi, Fethiye	High	82/2015	26.03.2015	36.502422	29.138055
B112	15.6	4.6	Absent	Kocada, Marmaris	Low	84/1985	11.04.1985	36.717138	28.020059
B113	15.3	5.2	Absent	Bozburun, Marmaris	High	80/2015	16.04.2015	36.633308	28.095443
B114	14.8	4.6	Present	Günlüklü, Dalaman	Medium	84/2015	28.03.2015	36.823459	28.797706
B115	15.5	4.6	Absent	Kızılada, Marmaris	Low	125/1986	17.05.1986	36.663620	28.034943
B116	14.8	3.5	Present	Uzunada, Marmaris	Low	89/1985	11.04.1985	36.718980	28.001515

B117	15.6	5.6	Absent	Tuzla Gölü, Milas	Medium	86/2015	11.04.2015	37.193422	27.588785
B118	17.5	7	Present	Çine Barajı	Medium	85/2015	13.04.2015	37.464142	28.146269
